# Widespread acquisition of antimicrobial resistance among *Campylobacter* isolates from UK retail poultry and evidence for clonal expansion of resistant lineages

**DOI:** 10.1186/1471-2180-13-160

**Published:** 2013-07-15

**Authors:** Helen ML Wimalarathna, Judith F Richardson, Andy J Lawson, Richard Elson, Richard Meldrum, Christine L Little, Martin CJ Maiden, Noel D McCarthy, Samuel K Sheppard

**Affiliations:** 1 Department of Zoology, University of Oxford, South Parks Road, Oxford OX1 3PS, UK; 2 Gastrointestinal Bacteria Reference Unit, PHE Colindale, 61 Colindale Avenue, NW9 5EQ, London, UK; 3 School of Occupational & Public Health, Ryerson University, 350 Victoria Street, Toronto M5B 2K3, Canada; 4 Institute of Life Science, College of Medicine, Swansea University, Swansea SA2 8PP, UK

## Abstract

**Background:**

Antimicrobial resistance is increasing among clinical *Campylobacter* cases and is common among isolates from other sources, specifically retail poultry - a major source of human infection. In this study the antimicrobial susceptibility of isolates from a UK-wide survey of *Campylobacter* in retail poultry in 2001 and 2004–5 was investigated. The occurrence of phenotypes resistant to tetracycline, quinolones (ciprofloxacin and naladixic acid), erythromycin, chloramphenicol and aminoglycosides was quantified. This was compared with a phylogeny for these isolates based upon Multi Locus Sequence Typing (MLST) to investigate the pattern of antimicrobial resistance acquisition.

**Results:**

Antimicrobial resistance was present in all lineage clusters, but statistical testing showed a non-random distribution. Erythromycin resistance was associated with *Campylobacter coli*. For all antimicrobials tested, resistant isolates were distributed among relatively distant lineages indicative of widespread acquisition. There was also evidence of clustering of resistance phenotypes within lineages; indicative of local expansion of resistant strains.

**Conclusions:**

These results are consistent with the widespread acquisition of antimicrobial resistance among chicken associated *Campylobacter* isolates, either through mutation or horizontal gene transfer, and the expansion of these lineages as a proportion of the population. As *Campylobacter* are not known to multiply outside of the host and long-term carriage in humans is extremely infrequent in industrialized countries, the most likely location for the proliferation of resistant lineages is in farmed chickens.

## Background

Campylobacteriosis is a major public health problem and is the most common bacterial cause of gastro-enteritis in the industrialised world [1]. *Campylobacter* is a commensal constituent in the microflora of a wide range of animals, and has been isolated from numerous hosts including domestic and wild mammals, birds and reptiles [2-4]. In humans, however, *Campylobacter* is pathogenic, routinely causing acute diarrhoea and occasionally serious sequelae including Guillain-Barre Syndrome and reactive arthritis [5]. The majority of human campylobacteriosis is caused by *C*. *jejuni* and *C*. *coli*[6]. Most cases are self-limiting and do not require therapeutic intervention but persistent or complicated cases and those affecting immuno-compromised patients, require antimicrobial treatment. Ciprofloxacin, a second generation fluoroquinolone, is commonly prescribed for the treatment of diarrhoea, especially in returning travellers, while macrolides are recommended where treatment is required for laboratory confirmed *Campylobacter*.

Since the late 1980′s there has been an observed increase in the incidence of resistance to antimicrobials, including fluoroquinolones and macrolides, in cases of human campylobacteriosis [7-11]. The development of resistance is often attributed to inappropriate or incomplete clinical usage of antimicrobials. However, this explanation is insufficient in the case of *Campylobacter* because most human cases are self-limiting and antimicrobial treatment is unusual. Furthermore person-to-person transmission is thought to be extremely rare in industrialised countries therefore there would be little opportunity for resistant lineages to proliferate [12]. With humans generally considered to be a dead-end host, there is a requirement to identify the most likely reservoirs for the acquisition of antimicrobial resistance in *Campylobacter*.

Contaminated chicken meat is among the major sources of *Campylobacter* associated with human disease. This has been demonstrated historically through risk assessment [13], case–control studies [14] and outbreak investigation [15,16], and through the 1999 ‘dioxin crisis’ natural experiment in Belgium, where all domestically produced poultry meat was withdrawn from sale and the incidence of human campylobacteriosis was reduced by 40% [17]. More recently attribution studies, using MLST, have been used to compare genotypes of *Campylobacter* strains carried by wild and farmed host animals with those in human disease. This has shown a link between strains found on chickens, retail poultry and those causing disease in humans [18-21].

This study quantifies the occurrence of antimicrobial resistance and investigates temporal trends among *C*. *jejuni* and *C*. *coli* isolates from retail poultry. By considering this in the context of a phylogeny for *C*. *jejuni* and *C*. *coli*, this study was designed to investigate the extent to which increases in antimicrobial resistance are the result of (i) widespread acquisition of resistance among dispersed *Campylobacter* lineages or (ii) clonal expansion of resistant lineages. This provides evidence for the location and nature of increased antimicrobial resistance among clinical *Campylobacter* strains.

## Results

Over the course of the study period a total of 194 STs, belonging to 27 clonal complexes (CCs), plus a further 82 STs not assigned to any recognised clonal complex were identified. Overall, the most abundant STs were ST 257 and ST 45, each representing 8.78% of the total sample, ST 827 (3.89%), ST 51 (3.19%), ST 21 (2.99%) and ST 573 (2.99%). There was no significant difference in the proportions of dominant STs between the two study periods.

**Figure 1:**
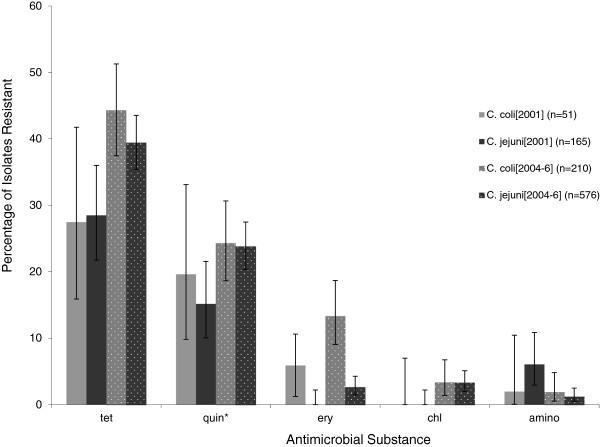
**Proportion of resistant isolates for each antimicrobial.** The percentage of resistant *C*. *coli* (light grey) and *C*. *jejuni* (dark grey) isolates are indicated for samples collected as part of UK retail poultry surveys in 2001 (solid colour) and 2004–5 (dotted). 95% confidence intervals, based on a binomial distribution (resistance: susceptibility) are given for tetracycline (tet), quinolones (quin), erythromycin (ery), chloramphenicol (chl) and aminoglycosides (amino).

Figure 1 presents the data for the percentage of resistant isolates of both *C*. *jejuni* and *C*. *coli* between the first phase of the study in 2001 and the second phase, in 2004–5. While there appears to be an increase in resistance to all of the tested antimicrobials between the two phases it was not possible to detect a statistically significant secular trend with a sample of this size.

Overall, 38.02% (95% CI 35.01 – 41.02) *C*. *jejuni* and *C*. *coli* isolates combined were resistant to tetracycline, 22.26% (95% CI 19.68 – 24.84) were resistant to quinolones, 4.59% (95% CI 3.29 – 5.89) were resistant to erythromycin, and 2.59% (95% CI 1.29 – 3.11) resistant to chloramphenicol.

**Figure 2:**
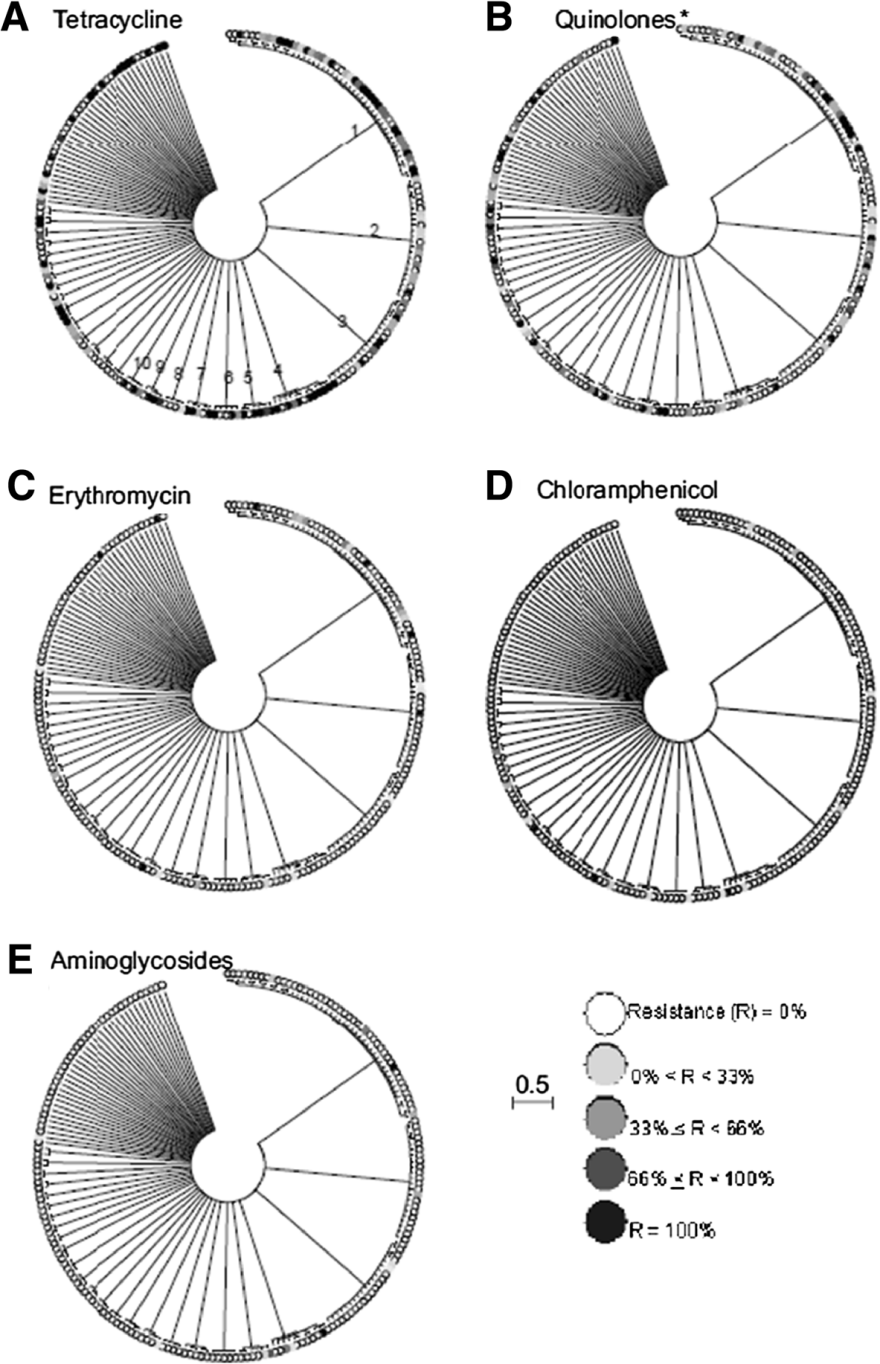
**ClonalFrame genealogies of *Campylobacter *isolates from UK retail poultry surveys in 2001 and 2004–5.** Grey-scale shading indicates the percentage of isolates in each ST with antimicrobial resistance to (**A**) tetracycline, (**B**) quinolones - naladixic acid & ciprofloxacin combined, (**C**) erythromycin, (**D**) chloramphenicol, (**E**) aminoglycosides. The scale bar indicates the genetic distance in coalescent units.

The genealogy estimated using ClonalFrame, applied to MLST data, showed a high degree of genetic structuring among retail poultry isolates (Figure 2), with many of the lineages frequently identified from clinical samples being represented. Isolate clustering on the tree correlated with previously identified clonal complex designations (Table 1). For four (tetracycline, quinolones, chloramphenicol & erythromycin) out of the five antimicrobial substances tested in this study, resistance phenotypes were dispersed throughout clusters of related lineages (Table 1). Nearly all isolates tested were sensitive to aminoglycosides, therefore this class of antimicrobial agent was excluded from further analyses.

**Table 1:** Number and percentage of isolates from each lineage that tested resistant to each antimicrobial

		**Number and percentage (%) of tested isolates resistant to antimicrobial substance**				
**LINEAGE (n)**	**Dominant CC**	**Tetracycline**	**Quinolones**^**3**^	**Erythromycin**	**Chloramphenicol**	**Aminoglycosides**
1 (209)	828	76 (36.4)	51 (24.40)	29 (13.88)	7 (3.35)	4 (1.91)
2 (187)	45	102 (54.55)	22 (11.76)	3 (1.60)	1 (0.53)	1 (0.53)
3 (131)	257	40 (30.53)	28 (21.37)	1 (0.76)	2 (1.53)	2 (1.53)
4 (44)	433	30 (68.18)	9 (20.45)	2 (4.55)	3 (6.82)	3 (6.82)
5 (21)	661	19 (90.48)	5 (23.81)	1 (4.76)	1 (4.76)	2 (9.52)
6 (16)	354	7 (43.75)	6 (37.50)	0	1 (6.25)	0
7 (7)	49	4 (57.14)	3 (42.86)	1 (14.29)	1 (14.29)	0
8 (5)	21	1 (20.00)	0	0	0	0
9 (35)	443	32 (91.43)	15 (42.86)	3 (8.57)	2 (8.57)	1 (2.86)
10 (5)	574	3 (60.00)	1 (20.00)	0	0	0
11 (8)	52	0	1 (12.50)	0	0	0
12 (3)	21	0	0	0	0	0
13 (11)	42	2 (18.18)	2 (18.18)	0	0	0
14 (12)	21	4 (33.33)	3 (25.00)	0	2 (16.67)	0
15 (21)	21	8 (38.10)	3 (14.29)	0	0	0
16 (3)	206	3 (100.00)	0	0	0	0
17 (4)	508	1 (25.00)	0	1 (25.00)	1 (25.00)	0
18 (10)	353	2 (20.00)	1 (10.00)	0	0	0
19 (10)	607	1 (10.00)	0	0	0	0
20 (7)	21	2 (28.57)	6 (85.71)	0	3 (42.86)	0
21 (4)	22	0	0	0	0	0
22 (7)	61	0	0	0	0	0
23 (10)		6 (60.00)	9 (90.00)	0	0	0
24 (3)		3 (100.00)	1 (33.33)	0	0	0
25 (2)		0	1 (50.00)	0	0	0

^1^ Lineages are defined as clusters of related genotypes based upon the ClonalFrame genealogy.

^2^ The clonal complex to which most of the STs belong.

^3^ Naladixic acid and ciprofloxacin.

A total of 22 out of the 25 multi-ST lineages contained isolates resistant to one or more antimicrobial. Tetracycline resistant isolates were present in 20/25 clusters, with the percentage of resistant isolates per cluster ranging from 10% to 100%. Isolates resistant to quinolone were present in 18/25 clusters and the proportion of resistant isolates ranged from 10% to 90%. Chloramphenicol and erythromycin resistant isolates were present in 11/25 and 8/25 clusters respectively and the proportion of resistant isolates per cluster did not exceed 42.9% in chloramphenicol or 25% in erythromycin.

For each antimicrobial, χ^2^ tests for homogeneity were carried out to test the null hypothesis that populations (species) are homogeneous in their resistance phenotypes. In the case of tetracycline, quinolones and chloramphenicol, p values > 0.1 were obtained, providing no evidence to reject the null hypothesis. In the case of erythromycin (p < 0.0005) there was a significant difference in the incidence of resistance between *C*. *jejuni* and *C*. *coli*, with erythromycin resistance being associated with *C*. *coli* (OR 6.52).

**Figure 3:**
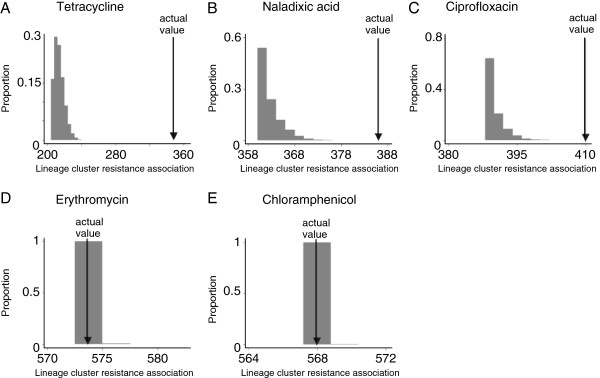
**Permutation test results for the association of lineage with resistance phenotype for the tested antimicrobials.** Comparison of a measure of association of resistant lineages with that expected by chance for (**A**) tetracycline, (**B**) naladixic acid, (**C**) ciprofloxacin, (**D**) erythromycin, (**E**) chloramphenicol. The arrows show the results from the data compared with frequency histograms of the scores from 10,000 permutations of the data which show the expected distribution of scores if no association exists. No comparison was made for aminoglycosides because too few isolates displayed resistance and so the test had no power.

Further, permutation tests were carried out for each antimicrobial, to test the null hypothesis that resistance was randomly distributed throughout the *C*. *jejuni* lineages. There was statistical support for some association between clade and probability of antimicrobial resistance for tetracycline and quinolones (naladixic acid and ciprofloxacin) in *C*. *jejuni*, although this is an incomplete explanation in itself. For erythromycin and chloramphenicol no statistical support for an association was identified (Figure 3).

## Discussion

From the clinical perspective the observed prevalence of resistance of *C*. *jejuni* and *C*. *coli* isolates to antimicrobial agents is high throughout the study period. These findings are consistent with published data from clinical *Campylobacter* isolates which show high levels of antimicrobial resistance over a comparable time period [22] and with other studies that show that antimicrobial resistance patterns in clinical strains closely resemble those observed in chicken meat isolates [23].

The high incidence of resistance to tetracycline in both *C*. *jejuni* and *C*. *coli* indicates that this drug would be of little use for the treatment of campylobacteriosis. In the 2004–5 study period the combined prevalence of quinolone resistance in *C*. *jejuni* and *C*. *coli* isolates was 23.9% (188/176 samples) while the prevalence of erythromycin (currently recommended for the treatment of laboratory-confirmed campylobacteriosis) resistance in *C*. *coli* was 13.3% (28/210 samples). These levels of resistance are likely to represent an unacceptable frequency of therapeutic failure of the drugs indicated for the treatment of human campylobacteriosis.

The high levels of antimicrobial resistance cannot be accounted for exclusively by high numbers of a particular group of resistant genotypes. Rather, there is evidence for widespread acquisition of resistance among relatively distantly related lineages from retail poultry. This is consistent with a small-scale study of *C*. *jejuni* isolated from chicken meat in Senegal where quinolone resistant phenotypes were present in three out of four tested lineages, and also dispersed throughout singleton STs [24]. It is possible that mutations that confer antimicrobial resistance have occurred in multiple lineages. However, bacteria can acquire genetic material, including antimicrobial resistance genes, from relatively distantly related lineages through horizontal gene transfer [25,26]. Horizontal Gene Transfer (HGT) can involve recombination between lineages, or acquisition of plasmids, which has been demonstrated to be the main mechanism of tetracycline resistance in *Campylobacter*[27]. There is also evidence that plasmid acquisition may mediate resistance to chloramphenicol and aminoglycosides [28,29]. Resistance to macrolides is conferred by a 2 bp change in the putative erythromycin binding site. Resistance to fluoroquinolones is most usually the result of a single mutation in the *gyrA* region [30]. The widespread antimicrobial resistance in the *Campylobacter* populations, is likely to be the result of horizontal gene transfer as well as multiple independent mutation events.

When conditions are such that antimicrobial resistance confers a strong selective advantage, lineages that trace ancestry to resistant isolates will increase as a proportion of the population [31]. Under these circumstances a phylogenetic tree will show clusters of resistant lineages that have expanded clonally. Consistent with this, statistical analyses of the ClonalFrame tree of retail poultry isolates indicated that resistant phenotypes were not randomly distributed but showed some clustering within lineages. At the highest level there was a species-specific association with erythromycin resistance correlated with *C*. *coli*, as in previous studies of *Campylobacter* in pigs, turkeys and chickens [32-35].

Resistance to tetracycline, quinolones and chloramphenicol showed no association with either *Campylobacter* species, but all were non-randomly distributed among *C*. *jejuni* lineages. This could indicate that antimicrobial resistance, having arisen in an ancestral lineage, is propagated clonally by vertical transmission. This finding is consistent with previous studies, in which associations were found between particular *C*. *jejuni* STs and serogroups, and a *gyrA* gene mutation which is a putative mechanism of resistance to quinolones [12].

For clonal expansion of resistant lineages to have occurred among isolates from retail poultry requires that strains had an opportunity to multiply. Mutation may occur stochastically but persistence is influenced by the fitness of organisms to compete in an environment containing antimicrobials. Human campylobacteriosis is self-limiting and person-to-person spread is thought to be rare, therefore while the human gut may be an antimicrobial rich environment, strains that acquire resistance are not propagated and are lost from the population. Retail poultry meat itself is an unlikely environment in which antimicrobial resistant strains increase as a proportion of the population because *Campylobacter* are not thought to multiply outside of the host. Isolates from retail poultry essentially represent a subset of those found in chickens on the farm and therefore resistance among these strains is likely to reflect resistance patterns among isolates inhabiting chicken guts [36,37].

Antimicrobials have historically been used in livestock farming both for the treatment of infections and as growth promoters. The practice of administering growth promoters containing antimicrobials analogous to those used in human medicine was banned in EU countries in 2003, and in 2006 the use of all antimicrobial growth promoters was banned in the EU [http://www.vmd.gov.uk/fsf/antimicrobial_agp.aspx]. However, specific antimicrobials are licensed for therapeutic use in poultry. These include danofloxacin and difloxacin from the quinolone and fluoroquinolone family, several tetracyclines, several macrolides (including two varieties of erythromycin), and a number of aminoglycosides. Amphenicols are not licensed for use in poultry farming in the UK. Previous studies have speculated that where flocks testing positive for *Campylobacter* and other infections are treated *en masse* through the water supply accurate dosing is impossible and an individual bird may receive a dose too low to inhibit bacterial growth completely, thereby favouring antimicrobial resistant strains [38]. Chickens may be considered a possible reservoir in which antimicrobial resistant *Campylobacter* may emerge. This has been shown in experimental conditions where resistance can be induced in *Campylobacter*-colonised chicken flocks, following treatment with fluoroquinolones [38,39].

## Conclusions

The findings of this study suggest that antimicrobial resistance in *Campylobacter* isolated from chicken meat is widespread and may be increasing. Since retail poultry is considered to be one of the most important reservoirs of human *Campylobacter* infections, this pervasive resistance is likely to have far-reaching public health consequences. The diffuse pattern of resistance suggests that horizontal gene transfer has a role in the acquisition of resistance and evidence for the proliferation of resistant lineage clusters indicates that conditions occur that favour resistant strains – potentially on poultry farms. As pressure to meet the demand for poultry has increased there has been a requirement for greater intensification of farming practices. The consequences of this are not fully understood but the trend towards increasing levels of antimicrobial resistance among *Campylobacter* isolates from retail poultry has implications for containing outbreaks of drug resistant strains in humans.

## Methods

### Retail poultry survey isolates

*Campylobacter* isolates (n = 1002) were obtained from the Health Protection Agency (HPA) Centre for Infections archive, comprising isolates from three UK retail chicken *Campylobacter* surveillance studies. Random, stratified samples of 214, 535 and 253 isolates were drawn from the National Retail Poultry Survey, April – June 2001; the Coordinated Local Authority Sentinel Surveillance (CLASSP) Study (2004–05); and Wales and Northern Ireland Surveillance Study (2001–06), respectively [40-42]. In total, 214 isolates from 2001 and 788 from 2004–05 were selected. The isolates represented both independent butchers and large multiple outlet retail chains. 75% of all isolates in the current study were of *C*. *jejuni*, and the remainder were of *C*. *coli*, and the sample was stratified to ensure that 50% of isolates were collected in England, and the remaining 50% were divided evenly between Northern Ireland, Scotland and Wales.

### Culture

All *Campylobacter* strains had been stored in the archive at −80°C in Microbank cryovials (Prolab PL1605/G) prior to subculturing on Columbia Blood Agar (CBA). Plates were incubated for 48 hours in a MACS-VA500 Variable Atmosphere Workstation (Don Whitley Scientific Ltd) under microaerobic conditions (5% CO_2_, 5% O_2_, 3% H_2_ and 87% N_2_) at 37°C. All microbiology procedures were performed according to the standards of the Clinical Pathology Accreditation (UK).

### Determination of antimicrobial resistance

All isolates were screened for antimicrobial susceptibility (no growth) or resistance (growth) by the breakpoint screening method [43]. Isolates were grown on Columbia Blood Agar for 24 hours prior to suspension in distilled water, with a density of bacterial cells equal to a Macfarlands 0.5 standard for inoculation of antimicrobial test plates. Individual antimicrobial substances tested were incorporated into separate Iso-Sensitest Agar, enriched with 5% horse blood, in the following concentrations: chloramphenicol 8 μg/ml; gentamicin 4 μg/ml; kanamycin 16 μg/ml; neomycin 8 μg/ml; tetracycline 8 and 128 μg/ml; nalidixic acid 16 μg/ml; ciprofloxacin 1 μg/ml; and erythromycin 4 μg/ml. The concentration of ampicillin tested changed from 32 μg/ml to 8 μg/ml during the course of the retail poultry surveys, thus ampicillin resistance was excluded from this study. A nationally or internationally recognised standard for the testing of antimoicrobial sensitivities in *Campylobacter* does not exist. The *Campylobacter* Reference Unit therefore developed and standardised a breakpoint method. While it differs from practices in some other laboratories it provides consistency within this dataset.

### DNA boilate preparation

Boilates for use as template in PCR reactions were prepared as follows. A cell suspension of each culture was made in 125 μl phosphate buffered saline or in water (Sigma Aldrich, UK) in a 0.2 ml PCR tube. Suspensions were vortexed and transferred to a heat block at 100°C for five minutes. This killed cell suspension was clarified by centrifugation at 13, 000 rpm for 10 min and stored at −20°C.

### PCR, Sequencing and bioinformatics

DNA template arrays were created in 96-well Thermo-fast®, polypropylene plates (Abgene, UK) and seven-locus MLST was carried out in Oxford by standard methods using published primers [40,44]. Each 25 μl PCR reaction comprised molecular grade water (Sigma-Aldrich, United Kingdom), 2.5 μl 10x PCR buffer (Qiagen Ltd.), 0.25 μM each of forward and reverse primer, 0.2 mM dNTP mix (Invitrogen Ltd.), 0.025 units/μl (0.125 μl) taq polymerase (Qiagen Ltd.) and 2 μl of template DNA. The PCR thermal cycle began with a 15 min denaturation step at 95°C, followed by 35 cycles of 94°C for 30 seconds, 50°C for 30 seconds and 72°C for 1 minute, with a final extension at 72°C for 5 minutes. 5 μl of PCR products were visualised with ultraviolet transillumination following electrophoresis at 200 V (10 min) on a 1% (w/v) agarose gel in 1x TAE buffer (1 mM EDTA, 40 mM Tris-acetate). The amplification products were purified by precipitation with 20% polyethylene glycol–2.5 M NaCl [41] and stored at −20°C. Nucleotide sequencing PCRs were performed in both directions with the same primers (f or r), diluted in water. Reactions were carried out in 10 μl volumes containing 2 μl of PEG precipitated DNA resuspended in water, 1.0 μl 5x buffer, 0.02 μl BigDye Terminator v3.1 mix (Applied Biosystems, UK) and 0.25 μM of either the forward or the reverse primer. Cycling parameters were as follows: 30 cycles of 96°C for 10 s, 50°C for 5 s, and 60°C for 2 min. Unincorporated dye terminators were removed by precipitation of the termination products with 95% ethanol, and the reaction products were separated and detected with an ABI Prism 3730 automated DNA sequencer (Applied Biosyststems, UK). Forward and reverse sequences were assembled from the resultant chromatograms using the Staden suite of computer programs from the Genetics Computer Group package (Madison, WI). The consensus sequence was queried against the *Campylobacter* database to give an allele number. The combination of alleles for the seven housekeeping genes gave the sequence type (ST). STs are assigned into genetically related clonal complexes, based on sharing four or more alleles with the central genotype.

A database was developed in the framework provided by the existing *Campylobacter* profiles database, [http://pubmlst.org/Campylobacter/] which covers the species *C*. *jejuni* and *C*. *coli* and is based on mlstdbNet software [42]. The molecular data on this database includes MLST and antigen sequence alleles.

### Data analysis

A phylogeny was estimated from the study data using ClonalFrame [45]. This model-based approach to determine bacterial microevolution distinguishes point mutations from imported chromosomal recombination events – the source of the majority of allelic polymorphisms. This allows more accurate estimation of clonal relationships. A 75% consensus cut-off was imposed, meaning that only branches identified in 75% or more of the sampled trees were used in the final consensus trees. The trees shown are consensus trees of 6 ClonalFrame runs each with a 1,000 burn in and 10,000 iterations. The strict parameters used to generate the consensus trees ensured that cluster membership was robustly supported.

Binomial exact 95% Confidence Intervals were calculated for the percentage of *C*. *coli* and *C*. *jejuni* isolates resistant to each antimicrobial in the first and second phases of the study to test for significant secular trends. χ^2^ tests were carried out, to test for homogeneity of resistance to each antimicrobial. The null hypothesis was that populations (species) are homogeneous in their resistance phenotypes. Permutation tests were then carried out for each antimicrobial to test the null hypothesis that there is no association between lineage and antimicrobial resistance phenotype within *C*. *jejuni*. Association between antimicrobial resistance and lineage in the observed data was summarised by an association score. This score was calculated by adding the absolute values for each lineage of the difference between the number of resistant and the number of susceptible isolates in that lineage. Resistance patterns were then randomised across the dataset and an association score estimated for this permuted dataset. This process was repeated 10,000 times and the observed score compared with the range of scores obtained by permutation.

## Competing interests

The authors declare that they have no competing interest.

## Authors' contributions

The study was conceived and designed by SS, NM and MM. Sampling and antimicrobial testing was carried out by JR, AL, RM, and CL. MLST was carried out by SS. Analysis was performed by SS, HW, and NM. The paper was written by HW, SS NM with contributions from the other authors. All authors read and approved the final manuscript.
